# Length of Stay of Emergency Department Patients with Stimulant Intoxication Receiving Intravenous Fluid

**DOI:** 10.5811/westjem.53133

**Published:** 2026-05-15

**Authors:** Kent C. Grimes, Brandon Dyer, Daniel Calkins, Teagen Smith, Heather Henderson

**Affiliations:** * University of South Florida, Morsani College of Medicine, Department of Emergency Medicine, Tampa, Florida; † University of South Florida, Morsani College of Medicine, Tampa, Florida; ‡ University of South Florida, Research Methodology and Biostatistics Core, Tampa, Florida

## Abstract

**Introduction:**

Intravenous (IV) fluids are routinely administered empirically in the emergency department (ED) for patients presenting with stimulant intoxication (eg, cocaine, methamphetamine, synthetic marijuana), although the literature is sparse regarding the benefits and risks of this practice. Our primary objective in this study was to assess whether empiric administration of IV fluids in the ED is associated with increased discharge length of stay (LOS) among ED patients presenting for stimulant intoxication who were subsequently discharged.

**Methods:**

This single-center, retrospective cohort study included 100 patients 18–69 years of age who were discharged from the ED with a non-incidental diagnosis related to stimulant intoxication between May 29, 2020–December 31, 2023, based on *International Classification of Diseases* code and chart review, in addition to a triage heart rate ≥ 90 beats per minute. We excluded patients if the medical decision-making reflected a clear indication for IV fluids or the presence of pre-defined confounding diagnoses or an uncontrolled factor that would have inherently impacted discharge LOS. Our primary outcome measure was discharge LOS. A multiple linear regression model controlled for the potentially confounding secondary outcome measures of age, sex, alcohol involvement, advanced imaging, sedation, and discharge escort.

**Results:**

A total of 100 patients were included, including 50 (50%) patients who did not receive IV fluids and 50 (50%) patients who did. Median patient age was 35 (interquartile range [IQR] 29–41) and 73% of patients were male. Patients who received IV fluids had a median LOS of 345 minutes (IQR 260–470) vs 305 minutes (IQR 205–413), with multivariable linear regression showing no statistically significant difference (β = 40.3, 95% CI, −13.6 to 94.2, *R*^2^ = 0.162).

**Conclusion:**

This study suggests that empiric IV fluid administration in stimulant-intoxicated ED patients was not significantly associated with discharge length of stay. Although the observed difference and confidence interval suggest the possibility of a clinically meaningful increase in discharge LOS with empiric IV fluid, these findings should be interpreted cautiously. Time is an important resource in high-volume ED settings, and this study suggest the need for judicious use of IV fluids in the absence of a clear indication.

## INTRODUCTION

In recently released guidelines regarding the treatment of patients with stimulant use disorder, the American Society of Addiction Medicine provided a general recommendation for supportive care in cases of stimulant intoxication (eg, cocaine, methamphetamine, synthetic marijuana) including the use of intravenous (IV) fluids, while also acknowledging a lack of evidence for more specific supportive measures.^1^ The risks and benefits of empiric IV fluid administration in the stimulant-intoxicated patient have not been well studied.^1^ For the purpose of this study, the administration of IV fluids without a clear indication and anticipated effect, such as in the case of volume resuscitation, will be referred to as empiric use of IV fluids.

*Tintinalli’s Emergency Medicine* suggests that intoxicated patients in general do not require fluid replacement unless they are experiencing fluid depletion or are at risk of rhabdomyolysis,^2^ but the textbook stops short of acknowledging potential harms from empiric IV fluids such as increased emergency department (ED) discharge length of stay (LOS)^3^ and, more importantly, pulmonary edema, hypoxia, and ultimately need for admission.^4^,^5^ Acute respiratory compromise may be an even greater risk in patients who smoke stimulants.^6^ These are extreme consequences of the sympathomimetic toxidrome, which may consist of tachycardia, hypertension, tachypnea, hyperthermia, sweating, mydriasis, and seizures, thereby often requiring close monitoring and at times sedating medications such as benzodiazepines in the initial period as part of supportive care.^1^

In the case of alcohol intoxication, empiric use of IV fluids does not increase the rate of intoxicant clearance^7^ and has either no effect on^8^,^9^ or increases^3^ discharge LOS. This remains controversial,^10^ particularly when there is clinical suspicion for hypovolemia.^11^ Hypothetical extrapolation of these findings from alcohol intoxication to stimulant intoxication provides a starting point in the effort to add to the medical literature. To our knowledge, this is the first study to specifically evaluate the association of empiric IV fluid administration on discharge LOS in stimulant-intoxicated patients in the ED.

### Importance

If the administration of empiric IV fluids in cases of stimulant intoxication without apparent indication were found to be associated with an increased discharge LOS, such a finding could lead to more judicious use of IV fluids, thereby decreasing resource use, cost of care, and risk of potential harm from IV catheter placement and fluid overload.

### Goals of This Investigation

Our primary objective in this study was to assess whether empiric IV fluid administration in the ED is associated with an increase in discharge LOS among ED patients presenting for stimulant intoxication who were subsequently discharged.

## METHODS

### Study Design and Setting

We conducted this retrospective cohort, observational study in an urban, tertiary-care ED in Tampa, Florida. The study was considered exempt from full review and was provided a HIPAA waiver by the University of South Florida Institutional Review Board (IRB) (STUDY007726).

### Selection of Participants

This study included patients 18–69 years of age discharged from the ED with a non-incidental diagnosis related to stimulant intoxication based on *International Classification of Diseases, 10^th^ Rev*, code (Appendix 1) and chart review. The study thus relied on real-time clinical decision-making of the treating physician to make the diagnosis based on clinical experience without a standardized protocol, in addition to a triage heart rate ≥ 90 beats per minute. Our intent was to attempt to decrease misclassification, particularly by excluding incidental or resolving stimulant intoxication.

**Population Health Research Capsule**

What do we already know about this issue?

*Intravenous fluids are commonly given to stimulant-intoxicated ED patients, but evidence regarding the benefits and risks of this practice is lacking*.

What was the research question?

*Is IV fluid use without a clear indication associated with increased discharge length of stay in stimulant-intoxicated ED patients?*

What was the major finding of the study?

*The IV fluid group had a 40-minute longer discharge length of stay (β = 40.3, 95% CI, −13.6 to 94.2, R^2^ = 0.162)*.

How does this improve population health?

*Findings support more judicious use of IV fluids, helping reduce unnecessary resource utilization and ED crowding in high-volume systems*.

Patients were excluded if their discharge diagnoses included laceration(s) requiring repair, bone fracture(s), dehydration, acute kidney injury, rhabdomyolysis, newly diagnosed infection, pregnancy, seizure, suicidality, decompensated chronic psychotic disease, or otherwise any clear indication in the documented medical decision-making as to why IV fluid was administered or the discharge LOS would be inherently prolonged or shortened. For example, we excluded charts if documentation included a patient temporarily refusing care; if difficult IV access was noted to cause a delay; and if delays in advanced imaging were noted, as well as other case-by-case situations that represented a delay in care and, therefore, a potential delay in discharge. Research assistants (RA) who collected the study data were instructed to avoid interpretation, such as of physical exam documentation (eg, “dry mouth”) or point-of-care ultrasound assessments, and instead rely on the clinician’s interpretation (eg, “dehydration”) to determine whether the clinician had a clear indication for IV fluid administration, thus allowing us to exclude that chart. A training document was used to prepare the RAs for this task; every datapoint was verified at least one additional time after initial collection.

We conducted an a priori power analysis for the primary analysis method of multiple linear regression with a power of 0.9 and alpha of 0.05. Recent ED average monthly metric data was used to determine a control discharge LOS of 360 minutes (6 hours) in patients not receiving IV fluids. Consensus among emergency attending and resident physicians determined that, based on their medical decision-making practice pattern, 30 minutes would be the assumed minimal clinically important difference, representing the effect size. An assumption was made that the standard deviation of 60 minutes would be equal within the two groups. Put in other words, we assumed that 68% of patients intoxicated with stimulants who did not receive IV fluids would have a discharge LOS of 5–7 hours, and a difference of ≥ 30 minutes between the groups’ averages would be clinically significant, leading us to seek a moderate effect size, or Cohen *f*^2^ of 0.15, in the power analysis, resulting in a minimum sample size of 41 per group, or 82 total, using PASS 2022 software (NCSS, LLC, Kaysville, Utah).

This calculation also takes into account an adjustment for an additional six independent variables. Patients were included in the study, based on chart review within the electronic health record (EHR), until the necessary sample size was achieved. A patient could only be included once, and only their most recent encounter was included. This resulted in recruiting eligible patients who visited the ED from May 5, 2020–December 31, 2023.

### Interventions

Patients were grouped by whether IV fluid was administered as determined by a review of orders during the visit. This was defined as the administration of any crystalloid fluid as either a bolus or infusion, regardless of the indication. If the order was discontinued, the order details were examined to determine whether IV fluids had been started at all; if they had been started, we considered them to have been administered.

### Outcomes

The primary outcome of this study was discharge LOS in minutes. Multiple additional datapoints were collected as suspected confounding variables with the understanding that they could be used as secondary outcome measures in hypothesis-generating post-hoc analyses. The presenting heart rate and systolic blood pressure were collected to calculate the shock index (former divided by the latter) and be used as surrogates of dehydration or hypovolemia under previously discussed guidelines, all of which are reasons for IV fluid administration regardless of intoxication status. The dichotomous variables of alcohol involvement, need for advanced imaging (eg, computed tomography, magnetic resonance imaging, ultrasound), and administration of sedating medication (eg, benzodiazepine, antipsychotic, antihistamine) were all suspected to be common occurrences that inherently increase discharge LOS. Lastly, because patients are more likely to be discharged sooner if they have a sober escort, we also collected this information.

### Measurements

Study investigators obtained all data points from the EHR by chart review and consolidated them in a datasheet, with a second investigator verifying appropriate inclusion and accuracy of data. Review included the text of the visit note of the treating emergency physician and the table of signed orders during that encounter. Particularly in the case of whether alcohol was involved or an escort was available, if the information was not clearly available in the chart, an assumption was made and the datapoint was marked as a “no.” Criteria previously described by Worster et al to improve the quality of chart review studies were used in this study, including description of abstractor training, case selection criteria, variable definitions, abstraction forms, performance monitoring, identification of the medical record, sampling methodology, missing-data management plan, and IRB approval.^12^

### Analysis

We summarized continuous variables as median (interquartile range [IQR]) and then compared them between groups using Mann-Whitney U tests. Categorical variables were summarized as frequency (percentage) and then compared between groups using chi-square tests. Additionally, we analyzed the association of discharge LOS and whether IV fluid was administered using a multiple linear regression analysis to support the continuous outcome variable while also controlling for the covariates of age, sex, alcohol involvement, advanced imaging ordered, use of sedating medications, and presence of an escort at discharge because of their potential contribution to the increase of discharge LOS, as was discussed in a similarly designed study.^8^ We assessed normality using a Shapiro-Wilk test and model fit was evaluated using *R*^2^. The analysis performed was prespecified in the study protocol, and we did not conduct additional analyses to limit the risk of overfitting. We performed all statistical analyses using IBM SPSS v29.0.2.0 (International Business Machines, Armonk, NY).

## RESULTS

### Characteristics of Study Subjects

We used the described chart review protocol to screen 1,397 charts within the study period, from which we selected 100 patients to include in the study sample: 50 (50%) patients who did not receive IV fluids and 50 (50%) patients who did receive IV fluids—a sample size that was achieved coincidentally based on the needs of a priori power analysis. The median patient age was 35 years (IQR 29–41), and 73% of patients were male. Table 1 shows the study participant characteristics, stratified by IV fluid administration status.

### Main Results

Based on a multiple linear regression model with discharge LOS as the dependent variable, IV fluid administration status as the independent variable, and the remaining variables listed in Table 2 as covariates, patients who presented to the ED with stimulant intoxication and received empiric IV fluid therapy had a median discharge LOS of 345 minutes (IQR 260–470) compared to patients with stimulant intoxication who did not receive IV fluids who had a median discharge LOS of 305 minutes (IQR 205–413), with multivariable linear regression showing non-statistical significance (*β = 40.3*, 95% CI, −13.6 to 94.2, *R*^2^ = 0.162). Datapoints for discharge LOS were found to be normally distributed (Shapiro-Wilk test *P* = .20), with a Q-Q plot that visually suggested normality as well. The full model summary is shown in Table 2.

Among the secondary outcomes, patients who received empiric IV fluids had a higher presenting heart rate (median 110 beats per minute [bpm] vs 105 bpm, *P* = .03) and were more likely to receive sedating medications (40% vs 20%, *P* = .03) compared to those who did not receive IV fluids. Additionally, advanced imaging being ordered had a significant positive correlation with discharge LOS (β = 76.7, 95% CI, 7.7–145.6), while male sex had a significant negative correlation with discharge LOS (β = −71.1, 95% CI, −130.5 to −11.7). The remaining covariates did not have any statistically significant association with status of IV fluid administration.

## DISCUSSION

### Summary and Interpretation of Main Results

In this single-center, retrospective cohort study, we found no statistically significant difference in discharge LOS of stimulant-intoxicated patients who received empiric IV fluids compared to those who did not. Although a priori power analysis was used to determine an appropriate sample size, the standard deviation within groups was higher than expected (149 minutes in the IV fluid group vs 124 minutes in patients who did not receive IV fluids, compared to the assumption of 60 minutes). Therefore, although this study may have ultimately been underpowered, the 40-minute (13%) higher discharge LOS and 95% CI indicating the possibility of up to 94 minutes (31%) longer discharge LOS among patients who received empiric IV fluid suggests that the true difference may be clinically significant. The model accounted for 16.2% of variance in discharge LOS (*R*^2^ = 0.162), a modest amount as to be expected given the multifactorial nature of ED throughput.

The secondary outcome result of the IV fluid administration group being more likely to have received sedating medications, with recognition of our inability to determine causality with our study design, may reflect an intention to replace insensible fluid losses in agitated, hyperthermic, or tachypneic patients who required sedation. Notably this difference of sedation administration between groups did not contribute significantly to a difference in discharge LOS in the regression model. Additionally, even despite controlling for diagnoses that would likely elicit fluid resuscitation, the finding that patients receiving empiric IVF fluids had a higher median presenting heart rate suggests that tachycardia alone may represent an indication for fluid resuscitation in some practices, although the difference between groups of 5 bpm lacks clinical significance.

This finding, combined with the fact that most patients in both groups had a normal shock index (median in both groups 0.77), post hoc analysis was deferred, and we limited vital signs to descriptive use. Considering the difference in presenting heart rate between groups, we conducted post hoc analyses in which the variables of presenting shock index, heart rate, and systolic blood pressure were added separately into the model (because of their substantial collinearity), with full results available in Appendix 2. These analyses produced similar results to the a priori analysis.

The secondary outcome of the association of advanced imaging ordered with increased discharge LOS (77 minutes) was expected, while other secondary outcomes—alcohol involvement, adminsitration of sedating medications, and presence of an escort at discharge—had nonstatistically significant effects in this study that was underpowered to detect them. The clinical significance of males having a statistically significant decrease in discharge LOS by 71 minutes compared to females is unclear but could reflect differences in perceived illness or bias in management and dispositioning.

### Comparison to Previous Studies

This study is novel in the context of stimulant use; however, similar designs have been used to evaluate the effect of empiric IV fluid administration on discharge LOS in alcohol-intoxicated patients which, arguably similarly, also showed no effect on^8^,^9^ or increases^3^ in discharge LOS. The potential for an efficacious dilutional effect from IV fluid administration has been challenged from the standpoint of discharge LOS, as previously discussed, in addition to biochemically in regard to intoxicant clearance in the case of alcohol.^7^

### Strengths

This study had multiple strengths. The use of an a priori power analysis prevented overfitting of an otherwise relatively simple chart review protocol. Additionally, we measured and controlled for potentially confounding variables based on prior studies with similar design, some of which were validated in this study. These features helped maintain internal validity and may improve generalizability of results to other urban centers. Overall, the study provides a pilot sample for future studies involving patients with stimulant intoxication.

Although not quantified in this study, there was an unexpectedly high number of patients who experienced symptoms of opioid intoxication, in many cases requiring naloxone to reverse respiratory depression, when they had intended to use a stimulant. While this quantification is likely variable between communities and has been studied,^13^ it may indicate a need for dedicated research from a public health perspective in an era of designer drugs containing multiple classes of substances.

### Clinical Implications

This study may help guide medical decision-making in environments where time and space are more limited. In practice, IV fluids should continue to be administered in stimulant-intoxicated patients when clinically appropriate, with the understanding that it may noticeably increase discharge LOS. Notably, the observed association of advanced imaging and increased discharge LOS in this study may shift attention toward this and other elements of care that more clearly affect throughput. These insights can inform departmental protocols and resource allocation strategies, particularly in high-volume EDs where efficiency is critical.

### Research Implications

Beyond discharge LOS, other areas of interest should be studied in the future that may build on this dataset, including determining factors that lead to increased oxygen requirements, intubation, and/or admission. Among those complicating factors, the route of stimulant administration may also have significance. The presence of other substances, particularly opioids, may also increase risk for negative outcomes. Further research on these topics would add to the relatively understudied area of stimulant intoxication.

## LIMITATIONS

We have identified several limitations that may affect the results, interpretation, and generalizability of the study. We aimed to exclude patients with a clear indication for IV fluid administration with diagnosis- and narrative-based exclusion criteria; however, it is possible that patients may have inadvertently been included or excluded if, for example, a diagnosis was not documented or went undetected, or an assessment of volume status was not documented. This is inherent to the retrospective design of the study. Similarly, the administration of prehospital IV fluids and sedating medications was not accounted for, nor was the infusion rate, volume, or type of IV fluids, although in most cases it consisted of 1–2 liter boluses of either normal saline or lactated Ringers solution.

Notably patients were not necessarily excluded from the non-IV fluid group if they had a contraindication to IV fluid administration (eg, current or high risk of volume overload). This increased study generalizability, however, may also have led to increased variance, although on subjective narrative review it was not a regular occurrence. Recall bias may also have influenced the results because the data point for alcohol involvement was at times determined based on the narrative documentation of a patient interview while they were intoxicated and could have resulted in misclassification bias, although in many cases blood alcohol levels were available.

Vital signs were collected and shock index calculated to be considered as surrogates for hypovolemia, but further exclusion accuracy could have been achieved through laboratory value interpretation such as kidney function, hemoconcentration, and urine studies. In many cases, no laboratory studies were ordered, and when significant abnormalities were present, they often were identified in the exclusion criteria through supporting diagnoses, such as acute kidney injury. The requirement of a heart rate ≥ 90 bpm was used as another surrogate means of increasing the likelihood that the patient in fact was intoxicated with a stimulant as is commonly the case; however, especially in the context of polysubstance use or even withdrawal, this surrogate marker may be misleading. For example, a patient who used stimulants and opioids and required naloxone for respiratory depression may be diagnosed, among other things, with stimulant intoxication. The addition of vital signs at discharge to the analysis could have provided more clarity between groups. Additionally, an attempt to elucidate a clear indication for IV fluid administration could have allowed for subgroup analyses with less variability; however, this would not have been reliable with the retrospective nature of the study, which instead addressed this by previously described exclusion criteria. Although the administration of IV fluids may expose patients to multiple adverse effects beyond an increase in discharge LOS,^4^–^6^ these events were not quantified in this study and often resulted in exclusion because of a requirement of admission; they represent an important area of future research.

Similarly, given the wide variety of stimulant intoxicants and their associated durations of effect in addition to dose and route of administration variability, the details of stimulant use were not quantified among the study groups and would likely be difficult to generalize to other centers, if even detectable, in the context of regional variability in drug supply. Based on urine drug screens and patient history, this study involved patients intoxicated with stimulants that included cocaine, methamphetamine, and synthetic marijuana; however, the exact intoxicant(s) from the local drug supply could not be confirmed with certainty in the clinical setting. Another potential contributing factor to variability was the lack of a standardized approach to the management of the stimulant-intoxicated patient, where the clinician’s practice pattern could have influenced discharge LOS significantly, although this study involved patients managed by dozens of different physicians over multiple years at a single center.

Finally, this study did not account for time of presentation or season of the year in addition to other commonly considered throughput modifiers such as peak volumes, variation of average acuity in the department, and general hospital throughput including severity of boarding of admitted patients in the ED having a ripple effect on resource demand. These limitations highlight the complexity of studying acutely intoxicated patients in the ED.

## CONCLUSION

This study suggests that empiric IV fluid administration in stimulant-intoxicated ED patients was not significantly associated with discharge length of stay. Although the observed difference and confidence interval suggest the possibility of a clinically meaningful increase in discharge LOS with empiric IV fluid administration, these findings should be interpreted cautiously given the study’s limitations. Time remains an important resource in high-volume ED settings, and the results of this study suggest the need for judicious administration of IV fluids in the absence of a clear indication.

## Supplementary Information



## Figures and Tables

**Table 1:** Patient characteristics and outcome data stratified by administration of intravenous fluids among 100 patients who were discharged after a presentation for stimulant intoxication.

Variable	Total (N = 100)	IV fluids (n = 50)	No IV fluids (n = 50)	*P* value
Age, median (IQR)	35 (29, 41)	33 (27, 42)	36 (31, 41)	.47
Sex – Male, n (%)	73 (73%)	36 (72%)	37 (74%)	.82
Discharge LOS (min), median (IQR)	328 (243, 420)	345 (260, 470)	305 (205, 413)	.17
Presenting SBP (mm Hg), median (IQR)	135 (124, 148)	139 (129, 152)	134 (123, 144)	.19
Presenting HR (bpm), median (IQR)	108 (98, 118)	110 (100, 120)	105 (97, 111)	.03
Presenting SI, median (IQR)	0.77 (0.71, 0.91)	0.77 (0.72, 0.92)	0.77 (0.70, 0.87)	.62
Alcohol involved, n (%)	32 (32%)	19 (38%)	13 (26%)	.20
Advanced imaging ordered, n (%)	20 (20%)	9 (18%)	11 (22%)	.62
Received sedation, n (%)	30 (30%)	20 (40%)	10 (20%)	.03
Escort at discharge, n (%)	8 (8%)	4 (8%)	4 (8%)	1.00

*IV*, intravenous; *IQR*, interquartile range; *LOS*, length of stay; *SBP*, systolic blood pressure; *HR*, heart rate; *SI*, shock index.

**Table 2:** Stratification of discharge length of stay (LOS) by multiple linear regression modeling assessing the association between discharge LOS and administration of intravenous fluids, controlling for the listed variables, among 100 patients discharged after presenting for stimulant intoxication.

Variable	Coefficient (β)	95% Confidence interval	*P* value
Intravenous fluids	40.3	(−13.6, 94.2)	.14
Age	2.3	(−0.4, 5.1)	.10
Sex – Male	−71.1	(−130.5, −11.7)	.02
Alcohol involved	18.8	(−39.9, 77.4)	.53
Advanced imaging ordered	76.7	(7.7, 145.6)	.03
Received sedation	35.1	(−23.8, 94.0)	.24
Escort at discharge	−9.0	(−107.5, 89.6)	.86
