# Supplementary material for: Length of Stay of Emergency Department Patients with Stimulant Intoxication Receiving Intravenous Fluid
Source: West J Emerg Med. 2026 May 15;27(3):669–75. doi: 10.5811/westjem.53133 (PMC13246206; doi:10.5811/westjem.53133)
Supplement: Supplementary file 1 [file wjem-27-669-s001.pdf]

**APPENDIX 1**

**ICD-10 Codes and Corresponding Diagnoses Used for Case Identification from Encounters**

| ICD-10 Code | ICD-10 Diagnosis Description |
|---|---|
| F15.10 | Other stimulant abuse, uncomplicated |
| F15.120 | Other stimulant abuse with intoxication, uncomplicated |
| F15.121 | Other stimulant abuse with intoxication delirium |
| F15.122 | Other stimulant abuse with intoxication with perceptual disturbance |
| F15.129 | Other stimulant abuse with intoxication, unspecified |
| F15.90 | Other stimulant use, unspecified, uncomplicated |
| F15.920 | Other stimulant use with intoxication, uncomplicated |
| F15.921 | Other stimulant use with intoxication delirium |
| F15.922 | Other stimulant use with intoxication with perceptual disturbance |
| F15.929 | Other stimulant use with intoxication, unspecified |
| T43.601A | Poisoning by unspecified psychostimulants, accidental (unintentional), initial encounter |
| T43.602A | Poisoning by unspecified psychostimulants, intentional self-harm, initial encounter |
| T43.604A | Poisoning by unspecified psychostimulants, undetermined, initial encounter |
| T43.651A | Poisoning by amphetamines, accidental (unintentional), initial encounter |
| T43.652A | Poisoning by amphetamines, intentional self-harm, initial encounter |
| T43.654A | Poisoning by amphetamines, undetermined, initial encounter |
| F14.10 | Cocaine abuse, uncomplicated |
| F14.120 | Cocaine abuse with intoxication, uncomplicated |
| F14.121 | Cocaine abuse with intoxication delirium |
| F14.122 | Cocaine abuse with intoxication with perceptual disturbance |
| F14.129 | Cocaine abuse with intoxication, unspecified |
| F14.221 | Cocaine dependence with intoxication delirium |
| F14.222 | Cocaine dependence with intoxication with perceptual disturbance |
| F14.229 | Cocaine dependence with intoxication, unspecified |
| F14.90 | Cocaine use, unspecified, uncomplicated |
| F14.920 | Cocaine use with intoxication, uncomplicated |
| F14.921 | Cocaine use with intoxication delirium |
| F14.922 | Cocaine use with intoxication with perceptual disturbance |
| F14.929 | Cocaine use with intoxication, unspecified |
| T40.5X1A | Poisoning by cocaine, accidental (unintentional), initial encounter |
| T40.5X2A | Poisoning by cocaine, intentional self-harm, initial encounter |
| T40.5X4A | Poisoning by cocaine, undetermined, initial encounter |

| | |
|---|---|
| T40.721A | Poisoning by synthetic cannabinoids, accidental (unintentional), initial encounter |
| T40.722A | Poisoning by synthetic cannabinoids, intentional self-harm, initial encounter |
| T40.724A | Poisoning by synthetic cannabinoids, undetermined, initial encounter |
| F19.10 | Other psychoactive substance abuse, uncomplicated |
| F19.920 | Other psychoactive substance use with intoxication, uncomplicated |
| F19.921 | Other psychoactive substance use with intoxication delirium |
| F19.922 | Other psychoactive substance use with intoxication with perceptual disturbance |
| F12.929 | Cannabinoid use, unspecified with intoxication, unspecified |
| T44.901A | Poisoning by unspecified drugs primarily affecting the autonomic nervous system, accidental (unintentional), initial encounter |
| T44.902A | Poisoning by unspecified drugs primarily affecting the autonomic nervous system, intentional self-harm, initial encounter |
| T44.904A | Poisoning by unspecified drugs primarily affecting the autonomic nervous system, undetermined, initial encounter |

**APPENDIX 2**

**Stratification of discharge length of stay (DCLOS) by multiple linear regression modeling in post-hoc analysis** assessing the association between DCLOS and IVF administration, controlling for the listed variables, among 100 patients discharged after presenting for stimulant intoxication.

**Acronyms:** IVF = intravenous fluids; DCLOS = discharge length of stay

| **Regression Model – Adding Presenting Shock Index (SI) Variable** | | | |
|---|---|---|---|
| **Variable** | **Coefficient (β)** | **95% Confidence Interval** | **p-value** |
| IVF | 38.7 | (-7.3, 397.8) | .16 |
| Age | 2.4 | (-0.4, 5.1) | .09 |
| Sex – Male | -67.6 | (-128.0, -7.2) | **.03** |
| Alcohol Involved | 15.7 | (-43.8, 75.2) | .60 |
| Advanced Imaging Ordered | 74.6 | (5.2, 144.0) | **.04** |
| Received Sedation | 36.2 | (-23.0, 95.4) | .23 |
| Escort at Discharge | -2.4 | (-103.1, 98.3) | .96 |
| Presenting SI | 68.6 | (-131.6, 268.9) | .50 |

R-square = 0.162

| Regression Model – Adding Presenting Heart Rate (HR) Variable | | | |
|---|---|---|---|
| **Variable** | **Coefficient (β)** | **95% CI** | **p-value** |
| IVF | 37.7 | (-17.7, 93.0) | .18 |
| Age | 2.4 | (-0.4, 5.2) | .09 |
| Sex – Male | -69.5 | (-129.6, -9.5) | **.02** |
| Alcohol Involved | 17.4 | (-41.8, 76.6) | .56 |
| Advanced Imaging Ordered | 76.5 | (7.3, 145.8) | **.03** |
| Received Sedation | 33.4 | (-26.3, 93.0) | .27 |
| Escort at Discharge | -5.0 | (-105.5, 95.5) | .92 |
| Presenting HR | 0.5 | (-1.6, 2.6) | .64 |

R-square = 0.164

| Regression Model – Adding Presenting Systolic Blood Pressure (SBP) Variable | | | |
|---|---|---|---|
| **Variable** | **Coefficient (β)** | **95% CI** | **p-value** |
| IVF | 42.0 | (-12.4, 96.2) | .13 |
| Age | 2.3 | (-0.4, 5.1) | .10 |
| Sex – Male | -69.4 | (-129.2, -9.6) | **.02** |
| Alcohol Involved | 17.3 | (-41.7, 76.4) | .56 |
| Advanced Imaging Ordered | 75.0 | (5.6, 144.4) | **.03** |
| Received Sedation | 38.4 | (-21.6, 98.4) | .21 |
| Escort at Discharge | -7.0 | (-106.1, 92.1) | .89 |
| Presenting SBP | -0.5 | (-1.9, 1.0) | .53 |

R-square = 0.166
